# Novel antiviral activity of baicalein against dengue virus

**DOI:** 10.1186/1472-6882-12-214

**Published:** 2012-11-09

**Authors:** Keivan Zandi, Boon-Teong Teoh, Sing-Sin Sam, Pooi-Fong Wong, Mohd Rais Mustafa, Sazaly AbuBakar

**Affiliations:** 1 Department of Medical Microbiology, Tropical Infectious Disease Research and Education Center (TIDREC), Faculty of Medicine, University Malaya, Kuala Lumpur, Malaysia; 2 Department of Pharmacology, Faculty of Medicine, University Malaya, Kuala Lumpur, Malaysia

**Keywords:** Infectious disease, Flavivirus, Antiviral, Dengue, Flavonoid, Baicalein

## Abstract

**Background:**

Dengue is a serious arboviral disease currently with no effective antiviral therapy or approved vaccine available. Therefore, finding the effective compound against dengue virus (DENV) replication is very important. Among the natural compounds, bioflavonoids derived mainly from plants are of interest because of their biological and medicinal benefits.

**Methods:**

In the present study, antiviral activity of a bioflavonoid, baicalein, was evaluated against different stages of dengue virus type 2 (DENV-2) replication in Vero cells using focus forming unit reduction assay and quantitative RT-PCR.

**Results:**

Baicalein inhibited DENV-2 replication in Vero cells with IC_50_= 6.46 μg/mL and SI= 17.8 when added after adsorption to the cells. The IC_50_ against DENV-2 was 5.39 μg/mL and SI= 21.3 when cells were treated 5 hours before virus infection and continuously up to 4 days post infection. Baicalein exhibited direct virucidal effect against DENV-2 with IC _50_= 1.55 μg/mL and showed anti-adsorption effect with IC_50_ = 7.14 μg/mL.

**Conclusions:**

Findings presented here suggest that baicalein exerts potent antiviral activity against DENV. Baicalein possesses direct virucidal activity against DENV besides its effects against dengue virus adsorption and intracellular replication of DENV-2. Baicalein, hence, should be considered for *in vivo* evaluation in the development of an effective antiviral compound against DENV.

## Background

Dengue is among the most widespread mosquito-borne diseases. It is endemic in many tropical and sub-tropical parts of the world and is rapidly spreading to other countries where the mosquito vectors, *Aedes aegypti* and *Aedes albopictus* are found [1,2]. Dengue infection is caused by dengue virus (DENV), a flavivirus belonging to the *Flaviviridae* family. There are four distinct DENV genotypes, DENV-1, DENV-2, DENV-3 and DENV-4. All four genotypes can cause a wide range of illnesses ranging from a mild febrile infection, self limited dengue fever (DF) to severe dengue hemorrhagic fever (DHF) and dengue shock syndrome (DSS). Dengue has resulted in many deaths with an annual estimate of 50 million deaths worldwide [3]. Currently there is no licensed vaccine for dengue and the development of vaccine has been very challenging due to the complexity of immune responses against dengue. A number of reports have suggested that there is a direct correlation between the amount of DENV present in the blood during the viremic phase and the severity of dengue [4]. Therefore, viral load reduction due to using effective antiviral may perhaps decrease the chance of severe dengue complications, DHF and DSS. There are a number of plant-derived compounds with potential antiviral activity [5-10]. These include, bioflavonoids, which are polyphenolic plant derivatives with many patented biological benefits including as antivirals [11-13]. We have previously reported that the flavonoids fisetin and quercetin exhibited anti-dengue virus activities [14,15]. Baicalein is a flavone (C_15_H_10_O_5_) commonly isolated from the root of *Scutellaria baicalensis*. *Scutellaria baicalensis* is one of the traditional Chinese medicinal herbs and is among the *Labiatae* family. Its roots have been used as medication for some diseases such as different infectious diseases, inflammations, hypertension and hyperlipidemia. Here we examined the anti-dengue virus properties of baicalein.

## Methods

### Flavonoid

Baicalein was purchased from Indofine Chemical Company (Indofine, NJ, USA) and the stock solution (20 mg/mL) was prepared in dimethyl sulfoxide (DMSO) (Sigma-Aldrich, St. Louis, MO, USA). Stock solution was aliquoted and stored at −20°C until needed. Stock solution was diluted with Eagle’s Minimum Essential Medium (EMEM, Gibco, NY, USA) and was filtered through a syringe filter with 0.2 μm pore size (Millipore, MA, USA) at the time of experiment.

### Cells and virus

C6/36 mosquito cell line and the African green monkey kidney cells (Vero) from American Type Culture Collection (ATCC) were used in this study. Both cell lines were propagated and maintained in EMEM supplemented with 10% fetal bovine serum (FBS) (Gibco, NY, USA). Vero and C6/36 cells were incubated at 37°C and 28°C, respectively. Dengue virus type-2 (DENV-2) New Guinea C strain (NGC) was propagated in C6/36 cells and harvested after cytopathic effects (CPE) were observed on day seven post infection. Virus stock titer was determined by foci forming assay (FFA) as previously described [14] and then aliquoted and stored at −70°C. During the time of virus propagation, the FBS concentration of the cell culture medium was reduced to 2%.

### Cytotoxicity assay

In this study, cytotoxicity of the baicalein was determined using MTT assay as previously described [16]. Briefly, monolayer of Vero cells were seeded in 96-well microplates and treated with different concentrations of baicalein in triplicates. Cells were treated for four days at 37°C, under similar condition and duration used for antiviral activity assay. At the end of the incubation period, 15 μl of MTT solution (Promega, WI, USA) was added to each well. The microplate was kept at 37°C for 4 hours followed by adding 100 μl of the solubilization solution/stop mix to each well. The optical density (OD) of the wells were measured at 570 using 96-well plate reader (TECAN, Mannendorf, Switzerland) and the dose–response curve was plotted using GraphPad Prism 5 (GraphPad Software Inc., San Diego, CA).

### Focus Formation Unit Reduction Assay (FFURA)

Antiviral activity of baicalein was determined by measuring the reduction in the number of viral foci. Briefly, confluent monolayers of Vero cells were prepared in 24 wells cell culture microplate. The infected cells were overlaid with 1.5% CMC containing EMEM with 2% FBS. Viral foci were visualized using peroxidase-based foci staining assay [17] four days post infection. The number of DENV-2 foci was counted using stereomicroscope and the titer of virus was expressed as Foci-Forming-Unit (FFU). The base-line for negative control (untreated infected cells) was the mean of the viral foci number ± SD in untreated wells. Then the percentage of foci reduction (RF %) compared to negative control was calculated as follows: RF(*%*) = (C-T) × 100/C.

Where, C is the mean of the number of foci for negative control well (without compound) and T is the mean of the number of foci in treated wells. The data from reduction in number of viral foci were verified and confirmed by quantitative RT-PCR (qRT-PCR).

### Dengue virus quantitative RT-PCR

DENV-2 RNA copy number was measured using the quantitative RT-PCR method as previously described with some modifications [18]. Briefly, intracellular and extracellular DENV-2 RNA was harvested from the DENV infected Vero cells. Viral RNA was extracted using RNA extraction kits (Qiagen RNA extraction kit and Qiagen RNeasy kit). The real-time RT-PCR assay was performed by adding 1 μl of extracted DENV RNA to the SensiMix SYBR green mixture (Quantace, Watford, United Kingdom) which contained 7.4 μl ddH2O, 10 μl 2X SensiMix One-Step, 0.4 μl 50X SYBR Green solution, 10 units of RNAse Inhibitor, 50 pmol of forward (DNF) and also reverse (D2R) primers [19]. All samples were assayed in triplicate. The amplification was performed using the DNA Engine Opticon system (MJ Research/Bio-Rad, Hercules, CA) with the following thermal cycling conditions: reverse transcription at 50°C for 30 min, initial denaturation at 95°C for 10 min, followed by 45 cycles of 95°C for 15 sec, 59°C for 30 sec and 72°C for 30 sec. Melting curve analysis was subsequently performed at temperature from 60°C to 98°C to verify the assay specificity. The absolute quantities of viral RNA in the samples were measured with a standard curve that was generated with a 5-fold serially diluted viral RNA extracted from DENV-2 virus inoculums of known titer. The standard curve consisted of 7 points of concentration ranging from 10^7^ to 10^3^ FFU/ml. Each concentration was assayed in triplicate (Additional file 1: Figure S1).

### Prophylactic and continuous treatment studies

Anti-viral activity of baicalein was examined in Vero cells using different concentrations at various time point of virus infection. Vero cells were used as these cells allowed for efficient DENV replication in contrast to human cells which were less permissive *in vitro*. To determine the prophylactic anti-dengue activity of baicalein, Vero cells were pre-treated with baicalein for 5 hours prior to infection. The treatment media were removed and the treated cells were washed twice with PBS. The cells were inoculated with 200 FFU of DENV-2 and incubated at 37°C for 1 h for virus adsorption. After 4 days of infection, antiviral activity was determined by reduction in foci numbers as described above. In separate experiment, cells were pre-treated with baicalein for 5 hours prior to infection and the compound was continuously present in the cell culture for 5 days until termination of the experiment.

### Anti-adsorption activity

The activity of baicalein against adsorption of DENV-2 to the Vero cells was measured by inoculating the confluent Vero monolayers in 24-wells cell culture microplate with 200 FFU of DENV-2 in the presence or absence of different concentrations of baicalein and incubated at 37°C for 1 hour for virus adsorption. Then the cells were washed by sterile PBS twice and overlayed with 1.5% CMC containing EMEM with 2% FBS. After 4 days of experiment, anti-adsorption activity of baicalein was determined by reduction in foci numbers as described above.

### Post-adsorption antiviral activity

Antiviral activity of baicalein against intracellular replication of DENV-2 was performed by treating Vero cells with baicalein after adsorption with 200 FFU of virus for 1 hour at 37°C and following washing with PBS to eliminate the unabsorbed viruses. Cells were treated with different concentrations of baicalein for 4 days. Antiviral activity was determined by viral foci reduction assay and qRT-PCR.

### Direct virucidal assay

Extracellular anti-dengue activity of baicalein was investigated by incubating DENV-2 suspension containing 200 FFU with equal volume of the different concentrations of baicalein for 1 h at 37°C. Then, Vero cells were infected with the treated viral suspension for 1 h at 37°C. Cells were washed with PBS to remove the unabsorbed viruses. Then the microplate was incubated at 37°C for 4 days. Antiviral activity was determined by the reduction in number of viral foci and qRT-PCR.

### Statistical analysis

The cytotoxic concentration of baicalein that reduced cells survival by 50% (CC_50_) and the inhibitory concentration the reduced the number of foci by 50% (IC_50_) were determined using GraphPad Prism for Windows, version 5 (Graph Pad Software Inc., San Diego, CA, 2005). Selectivity Index value (SI) was determined as the ratio of CC_50_ to IC_50_ for that compound as previously described [14].

## Results

### Cytotoxicity of baicalein

**Figure 1:**
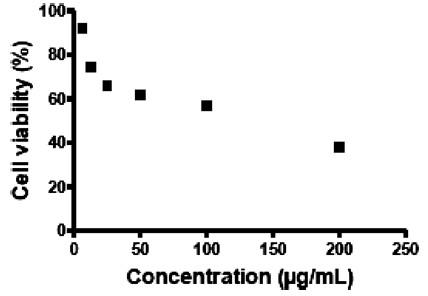
**Cytotoxicity of baicalein on Vero cells.** MTT assay was used to evaluate the cytotoxicity of the flavonoid. All experiments were conducted in triplicates.

Cytotoxicity of baicalein on Vero cells were evaluated using the MTT assay. The related CC_50_ was then calculated using Graph Pad Prism for Windows, version 5 (GraphPad Software Inc., San Diego, CA). Our results suggest that baicalein when added to Vero cells exhibited cytotoxic effects with CC_50_=109 μg/mL (Figure 1). More than 75% of Vero cells were viable in the presence of 12.5 ug/mL of baicalein which is important for our antiviral assays in next step. The vehicle control, 1% DMSO, did not show any cytotoxic effects against Vero cells.

### Antiviral assays with baicalein against DENV-2

**Figure 2:**
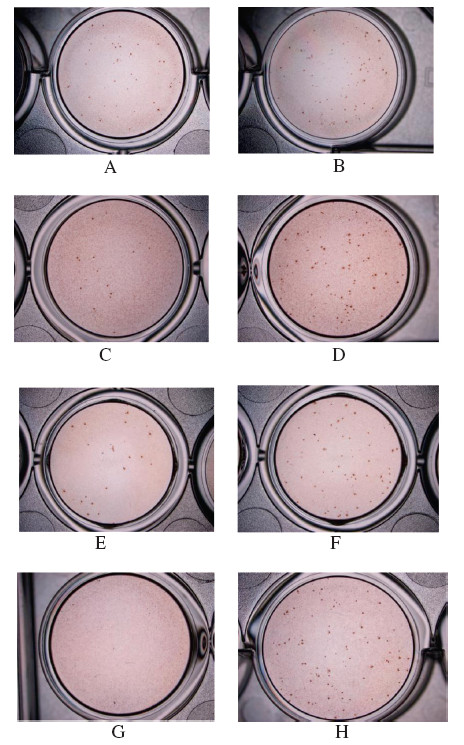
**DENV-2 foci in Vero cells treated with or without baicalein.** prophylactic treatment with 12.5 μg/ml of baicalein (**A**) or without baicalein (**B**); during adsorption with 12.5 μg/ml of baicalein (**C**) or without baicalein (**D**); after virus adsorption with 12.5 μg/ml of baicalein (**E**) or without baicalein (**F**); direct virucidal activity with 12.5 μg/ml of baicalein (**G**) or without baicalein (**H**). Foci were detected using dengue hyperimmune sera after 4 days of incubation.

Foci forming unit reduction assay was used to evaluate the *in vitro* anti-dengue virus activities of baicalein (Figure 2). Baicalein was added at the different phases of viral infection: i) pre-treatment for 5 hours prior to infection for its prophylactic activity, ii) treatment during virus adsorption time iii) treatment for 4 days post-adsorption, iv) continuous treatment from 5 h before infection and up to 5 days of infection and v) directly to cell free virus suspension to examine its direct virucidal effect.

**Figure 3:**
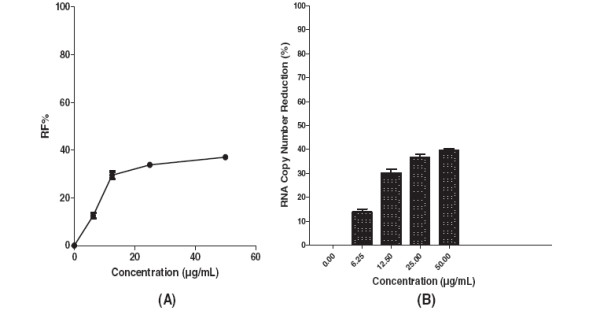
**Prophylactic effects of baicalein against DENV-2 replication.** Foci forming unit reduction assay was used to evaluate the prophylactic activity of the baicalein (**A**). and the DENV-2 RNA copies were quantified using qRT-PCR (**B**). All experiments were conducted in triplicates. The percentages of foci reduction (%RF) and RNA copy number reduction were obtained by comparing against the controls maintained in parallel.

**Figure 4:**
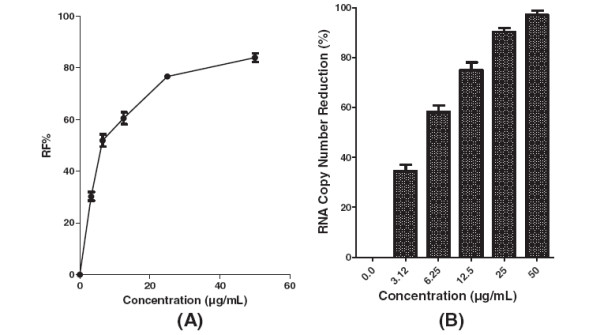
**Effects of baicalein against DENV-2 host cell adsorption.** Foci forming unit reduction assay was used to determine the anti-adsorption activity of baicalein against dengue virus (**A**) the respective DENV-2 RNA copies were quantified using qRT-PCR (**B**). All experiments were conducted in triplicates. The percentages of foci reduction (%RF) and RNA copy number reduction were obtained by comparing against the controls maintained in parallel.

**Figure 5:**
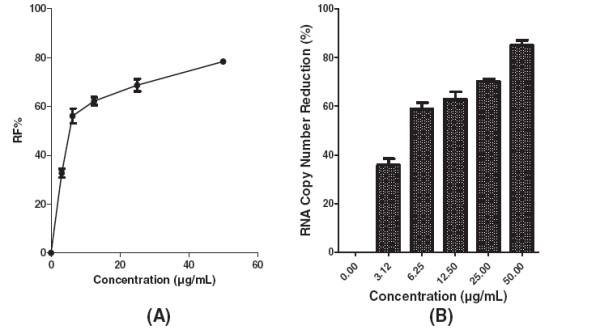
**Antiviral activity of baicalein against DENV-2 intracellular replication.** Foci forming unit reduction assay was used to evaluate the anti-dengue activity of baicalein after virus adsorption to the Vero cells **(A)** and the respective DENV-2 RNA copies were quantified using qRT-PCR **(B)**. All experiments were conducted in triplicates. The percentages of foci reduction (%RF) and RNA copy number reduction were obtained by comparing against the controls maintained in parallel.

Pre-treatment of Vero cells with 12.5 μg/mL decreased formation of virus foci to below 30% (Figure 3A). The base-line value for the negative control of prophylactic treatment was 76.6 ± 4 based on the mean of viral foci in un-treated wells. However, using the negative control base-line value the percentage of the foci reduction (RF %) was calculated and used for plotting. The level of DENV-2 RNA was reduced by 32% ± 0.8 at 12.5 μg/mL of baicalein when compared to the non treated DENV infected cells (Figure 3B). Baicalein when added simultaneously with the virus showed anti-adsorption activity against DENV-2 with IC_50_ = 7.14 μg/mL (Figure 4A) and with SI value of 16.1. The base-line mean value of viral foci in the negative control (un-treated wells) of anti-adsorption activity was 78.6 ± 2. Production of viral RNA decreased by 75% ± 3 in the presence of 12.5 μg/mL baicalein during the viral adsorption period (Figure 4B). In post adsorption assay, baicalein exhibited potent antiviral activity against DENV-2 with IC_50_ = 6.46 μg/mL (Figure 5A). The base-line value for the negative control of post-adsorption treatment was 69.6 ± 3.5 based on the mean of viral foci number in un-treated wells. The copy number of viral RNA decreased by 62.9% ± 3 when the infected cells were treated with 12.5 μg/mL (Figure 5B).

**Figure 6:**
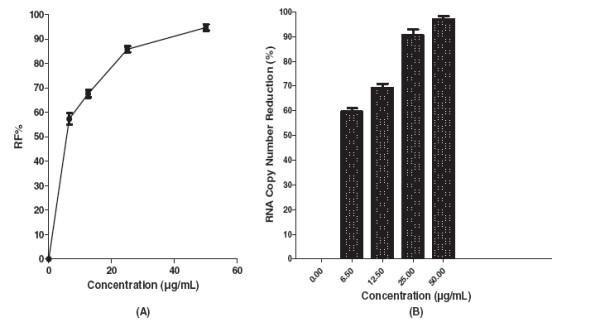
**Anti-dengue effect of continuous treatment with baicalein.** Foci forming unit reduction assay was used to evaluate the in vitro anti-dengue virus activities of the baicalein (**A**). The respective DENV-2 RNA copy number was quantified using qRT-PCR (**B**). The percentages of foci reduction (%RF) and RNA copy number reduction were obtained by comparing against untreated controls maintained in parallel.

The IC_50_ value for baicalein for continuous treatment of cells from 5 h before virus infection up to 4 days post infection was 5.39 μg/mL and its SI value was 21.3 (Figure 6A). The mean of viral foci number in negative control wells was 71 ± 3.5 as a base-line for the foci reduction percentage calculation. Quantitative RT-PCR results showed that 12.5 μg/mL of baicalein decreased the DENV-2 RNA production 69.2 ± 1.6 but at 25 μg/mL of baicalein viral RNA level production decreased more than 90% (Figure 6B).

**Figure 7:**
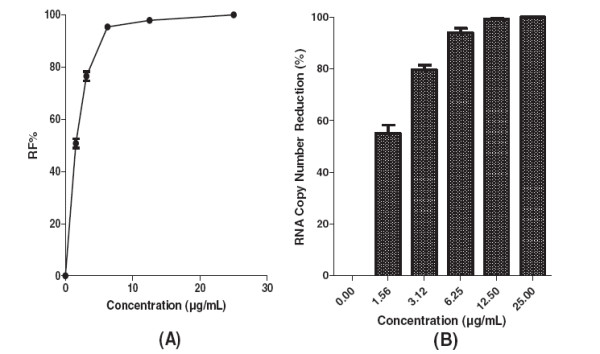
**Direct virucidal activity of baicalein against DENV-2.** Foci forming unit reduction assay was used to determine the direct anti-dengue virus activity of the baicalein. Baicalein was added directly to virus inoculum prior to infecting cells (**A**) and the respective DENV-2 RNA copies were quantified using qRT-PCR (**B**). All experiments were conducted in triplicates. The percentages of foci reduction (%RF) and RNA copy number reduction were obtained by comparing against the controls maintained in parallel.

Results from direct virucidal activity assessment of baicalein showed that baicalein exhibited a potent extracellular anti-DENV-2 activity with IC_50_ = 1.55 μg/mL (Figure 7A). The base-line value for the negative control of post-adsorption treatment was 82 ± 1 based on the mean of viral foci number in un-treated wells. Similarly, qRT-PCR analysis showed that 12.5 μg/mL of baicalein decreased the DENV-2 RNA production 99.2% ± 0.4 (Figure 7B).

## Discussion

Flavonoids in general are natural compounds that are ubiquitously found in plants. They are described as less toxic in comparison to other plant compounds such as the alkaloids and therefore, can be consumed in higher amount. Many studies have shown that flavonoids can have beneficial effects on human health and these ranges from anticancer to antimicrobial effects [20,21]. Several studies have also shown that flavonoids exerted antiviral activities against a number of common viruses including hepatitis B, herpes simplex viruses, human cytomegalovirus and few others [22-25]. There are also reports of inhibition of virus replication cycle by flavones, a subgroup of flavonoids that include compounds such as wogonin, apigenin, luteolin and baicalein [5,26,27]. A number of flavonoids have also been reported to exhibit significant anti-dengue properties [28-30]. More recently, we showed that flavones, more exactly, flavonols, fisetin and quercetin exhibited significant anti-dengue virus activities *in vitro* in contrast to hesperetin, naringenin, daidzein, naringin and rutin which showed no or only limited activities against DENV replication [14,15]. Baicalein is a flavone and the main natural resource for this compound is the roots of *Scutellaria baicalensis* a traditional Chinese medicine with different biological activities including antiviral activity [31]. Indeed, it has been reported that a flavone from the roots of *S.baicalensis* exhibited significant antiviral activity against influenza viruses after virus adsorption to the susceptible cell line [32]. Here, we demonstrated that another flavonoid, namely, baicalein exhibits significant inhibitory activity against DENV-2 replication in Vero cells. It is the most potent flavonoid against DENV identified thus far in comparison to fisetin and quercetin. Baicalein similar to other flavones share common 2-phenylchromen-4-one (2-phenyl-1-benzopyran-4-one) backbone except for the additional tri hydroxyl on the flavone (5,6,7-Trihydroxy-2-phenyl-chromen-4-one or 5,6,7-trihydroxyflavone).

Results from our study suggest that baicalein exhibits potent *in vitro* antiviral activity against all stages of the DENV-2 replication cycle such as adsorption stage (SI = 16.1) and intracellular virus replication (SI = 17.8). In addition, baicalein showed highly potent direct virucidal activity (SI = 74.3). Its prophylactic activity against DENV-2 replication was also observed, albeit weaker (SI = 1.06). These observations suggest that one of the possible mechanisms for baicalein extracellular and intracellular activities against DENV-2 could be attributed to its ability to bind and/or to inactivate important structural and/or non structural protein(s) of DENV-2. Such inhibitory mechanism was previously reported for pinostrobin which inhibits dengue virus NS3 protein [29]. Baicalein has also been reported to bind to HIV-1 integrase [33]. Furthermore, increasing SI values from 17.8 to 21.3 was observed when baicalein was used to treat cells 5 h before infection and continuously for 4 days post infection. In some studies, activity of several flavonoids against cellular RNA polymerases and formation of the complex with RNA were reported [34,35]. Therefore, it is possible that baicalein inhibits DENV replication by interfering with DENV-2 RNA polymerase and/or bind to the viral RNA which should be noted for future investigations. Results from qRT-PCR supported the findings from viral foci reduction but the inhibition of RNA production was more significant than inhibition of DENV-2 foci formation. In all our experiments, we showed that 0.5% of DMSO the solvent need initially to dissolve bioflavonoid, did not exhibit any antiviral activity against DENV-2 and this eliminated any probable antiviral activity from DMSO.

**Figure 8:**
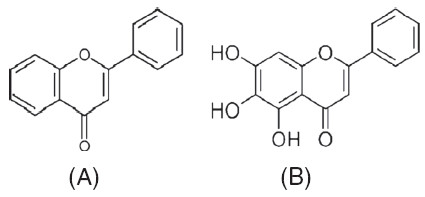
Chemical structure of flavone (A) and baicalein (B).

The specific active structure of baicalein that contributes to the inhibitory effects is not presently known. In an earlier study however, we showed that flavone significantly enhances DENV replication in Vero cells [36]. The differences in structure between these two compounds lies in the presence of three hydroxyl group in baicalein (Figure 8B) in comparison to the flavone (Figure 8A), suggesting the potential importance of the hydroxyl groups. This possibility is supported by the findings of a previous study that has demonstrated the presence and the positions of hydroxyl group in baicalein is crucial for its activity against HIV-1 reverses transcriptase enzyme in comparison to flavone [37].

## Conclusions

In summary, our study demonstrated that baicalein exhibited potent anti-dengue activity *in vitro*, in particular its virucidal activity against extracellular DENVs. Results from this study warrant future *in vivo* anti-viral, toxicity and pharmacokinetic studies as part of the developmental process for development of baicalein as potential anti-dengue therapeutic.

## Competing interests

The authors declare that they have no competing interests.

## Authors’ contributions

KZ participated in the design of study and carried out the antiviral and cytotoxicity studies and drafted the manuscript. BTT carried out the dengue virus propagation and titration. SSS participated in designing and performing the quantitative RT-PCR. MRM participated in study design and provided baicalein. WPF participated in the design of the study, performed statistical analyses and edited the manuscript. SAB conceived the whole study and edited the manuscript. All authors read and approved the final manuscript.

## Pre-publication history

The pre-publication history for this paper can be accessed here:

http://www.biomedcentral.com/1472-6882/12/214/prepub

## Supplementary Material

Additional file 1 **Figure S1.** Q-RT-PCR-Standard Curve Optimization. Click here for file
