# Supplementary material for: Novel antiviral activity of baicalein against dengue virus
Source: BMC Complement Altern Med. 2012 Nov 9;12:214. doi: 10.1186/1472-6882-12-214 (PMC3528482; doi:10.1186/1472-6882-12-214)
Supplement: Additional file 1 — Figure S1. Q-RT-PCR-Standard Curve Optimization. [file 1472-6882-12-214-S1.pdf]

	Type	Label	C(T)	ffu/mL
A1	Standard	Neat	14.32007	1.26E+08
A2	Standard	Neat	17.15083	1.26E+08
A3	Standard	Neat	13.88489	1.26E+08
B1	Standard	-1	14.60138	2.52E+07
B2	Standard	-1	16.34198	2.52E+07
B3	Standard	-1	15.65227	2.52E+07
C1	Standard	-2	16.79007	5040002
C2	Standard	-2	17.3835	5040002
C3	Standard	-2	17.28369	5040002
D1	Standard	-3	19.0136	1008000
D2	Standard	-3	19.0427	1008000
D3	Standard	-3	19.27902	1008000
E1	Standard	-4	21.95963	201600
E2	Standard	-4	22.06662	201600
E3	Standard	-4	21.668	201600
F1	Standard	-5	24.61732	40320.02
F2	Standard	-5	27.14153	8063.998
F3	Standard	-5	27.79537	8063.998
G1	Standard	-6	27.51627	8063.998
G2	Standard	-6	24.95085	40320.02
G3	Standard	-6	24.65402	40320.02
H1	Standard	-7	30.246	1612.8
H2	Standard	-7	29.72801	1612.8
H3	Standard	-7	29.9452	1612.8

virus stock=108 | 5-fold dilution

Template amount (ffu/ml)	Log template amount	Ct 1	Ct 2	Ct 3	Mean Ct
1.26E+08	8.100370545	14.32007	17.15083	13.88489	
2.52E+07	7.401400541	14.60138	16.34198	15.65227	15.12683
5040002	6.702430709	16.79007	17.3835	17.28369	17.15242
1008000	6.003460532	19.0136	19.0427	19.27902	19.11177
201600	5.304490528	21.95963	22.06662	21.668	21.89808
40320.02	4.605520739	24.61732	24.95085	24.65402	24.74073
8063.998	3.906550411	27.14153	27.79537	27.51627	27.48439
1612.8	3.207580515	30.246	29.72801	29.9452	29.97307

Standard Curve

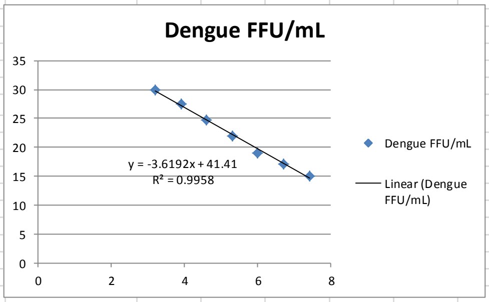

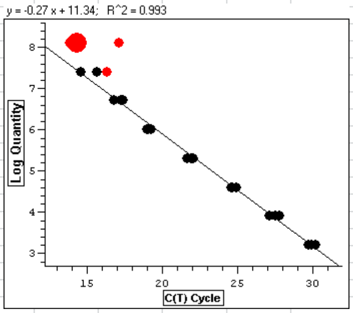

0.276304156 | 0.889314 Efficiency

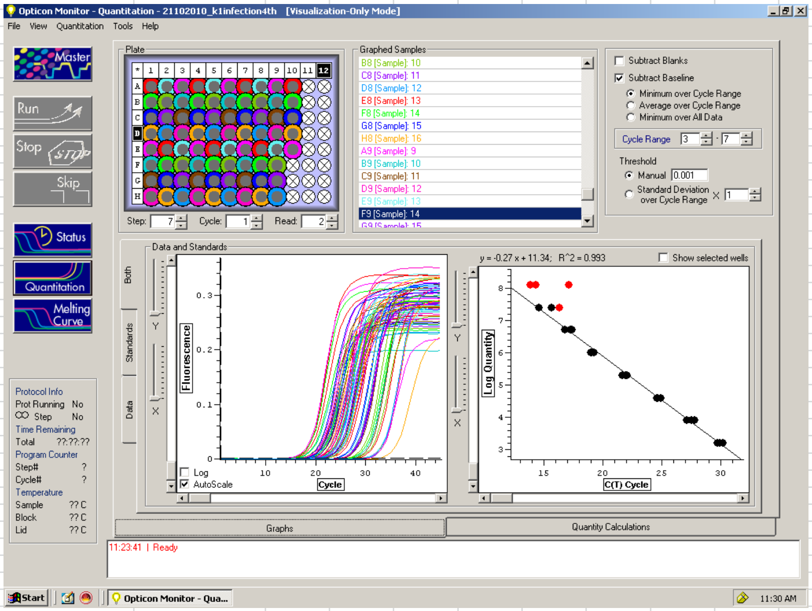

Q-RT-PCR-Standard Curve Optimization
